# The Effect of Radiofrequency Ablation of Posterior Nasal Nerves on Inflammatory Cytokines, Peak Nasal Inspiratory Flow, and Subjective Symptom Scores in Chronic Rhinitis: A Prospective Clinical Trial

**DOI:** 10.1177/00034894261449672

**Published:** 2026-05-09

**Authors:** David Z. Allen, Elena E. Skaribas, Ashwini Bandi, Sameer Siddiqui, Panayiotis Kontoyiannis, Jerril Jacob, Ella Hawes, Sun Hua, Kunal Shetty, William Yao, Martin J. Citardi, Amber U. Luong

**Affiliations:** 1 Department of Otorhinolaryngology-Head and Neck Surgery, McGovern Medical School, Houston, TX, USA; 2 McGovern Medical School, University of Texas Health Science Center, Houston, TX, USA; 3 Center for Immunology and Autoimmune Diseases, The Brown Foundation Institute of Molecular Medicine for the Prevention of Human Diseases, McGovern Medical School, The University of Texas Health Science Center at Houston, TX, USA

**Keywords:** radiofrequency ablation, rhinitis, chronic rhinitis, rhinorrhea, cytokines, posterior nasal nerve

## Abstract

**Background:**

Chronic rhinitis (CR) is inflammation of the nasal mucosa, causing a multitude of symptoms. Temperature-controlled radiofrequency neurolysis (RFN) of the posterior nasal nerve (PNN) is a treatment for CR. In allergic rhinitis (AR), Type 2 cytokines are drivers of symptom severity. This study explores whether RFN’s symptomatic improvements are linked to changes in nasal airflow and local cytokine levels.

**Methods:**

This prospective, single-arm study was conducted at the Texas Sinus Institute at The University of Texas Health Science Center at Houston. Patients with ≥6 months of CR symptoms, a reflective total nasal symptom score (rTNSS) ≥6, and refractory to medical management were included. Follow-ups occurred at 4 and 12 weeks, with the primary endpoint at 12 weeks. Symptom improvement, peak nasal inspiratory flow (PNIF) and cytokine changes were assessed.

**Results:**

Seventeen patients were enrolled with 15 patients undergoing the treatment. The procedure was well tolerated with no major adverse events. The rTNSS improved from a median of 7.5 to 4 at 12 weeks (*P* < .01). Median NOSE scores improved from 52.5 to 17.5 at week 12 (*P* < .05). Minimal clinically important differences (MCID) were achieved by 67% of patients for rTNSS and 50% for NOSE. There were no significant changes in PNIF. Patient-level change in IL-10 was significantly associated with improvement in NOSE score (*r* = −.70, *P* = .011).

**Conclusions:**

RFN of the PNN is a safe, effective in-office treatment for CR. While PNIF and group-level cytokines were not consistent indicators of improvement, the correlation between IL-10 and symptom relief suggests a potential link between neural ablation and the local mucosal microenvironment. This investigation shows the feasibility of cytokine monitoring to further investigate the mechanisms of rhinitis treatment.

## Introduction

Allergic rhinitis (AR) is mediated by an IgE-related response while non-allergic rhinitis (NAR) pathophysiology is linked to multiple triggers.^1^ The presentations of both are similar (both lead to symptoms of congestion and rhinorrhea, the hallmark symptoms of chronic rhinitis (CR)), and treatment consists of intranasal medications which often fail necessitating further treatment.^2-4^ Radiofrequency neurolysis (RFN) of the posterior nasal nerve (PNN), a terminal branch of the vidian nerve, is utilized to reduce hypersecretion.^5^ The NEUROMARK^®^ system from Neurent Medical (Neurent, Ireland) is approved for treatment of CR; this device delivers low level radiofrequency energy to tissues while limiting the total energy delivery via an impedance feedback loop.^6^ However, the efficacy of treatment is limited by subjective outcomes which are prone to placebo effect.^7,8^ As such, objective measurements such as peak nasal inspiratory flow (PNIF) or cytokines to evaluate disease burden are important. PNIF is a measure of airflow through the nasal cavities and is correlated with rhinitis.^9-11^ Type 2 cytokines are linked to AR and have not been explored after PNN treatment.^4^ Importantly, the local cytokine environment has been shown to be associated with rhinitis symptomatology.^12^ The aim of this investigation was to evaluate symptoms, PNIF, and cytokine changes after RFN of the PNN. One of the secondary objectives of this analysis was to determine the feasibility of cytokine measurement for in-office based rhinological procedures, of which there are few reported studies in the literature.

## Methods

This was a prospective, single-arm, single-institution study to evaluate the response to the Neuromark™ system in CR patients. Enrollment was conducted at The University of Texas Health Science Center at Houston with approval from The University of Texas Health Science Center at Houston Institutional Review Board.

Participants were eligible if they had CR symptoms for at least 6 months, poor response to medical management, and a Reflective Total Nasal Symptom Score (rTNSS) score ≥6, with ≥2 for rhinorrhea, and ≥1 for congestion. Exclusion criteria included active sinusitis, rhinitis medicamentosa, epistaxis, immunodeficiency, or prior surgery.

Prior to the procedure, rTNSS, nasal obstruction symptom evaluation (NOSE), PNIF, and nasal secretions were collected. Leukosorb paper (Pall Corporation, New York) was used for cytokine analysis which was placed within the middle meatus for 5 minutes and secretions were eluted by centrifugation, the values were averaged across both nostrils for analysis. Neurent Medical’s Neuromark™ device system (Generation 2.0) was used which consists of an in-office, disposable impedance-controlled RFN. The subjects had follow-up appointments scheduled at 4 and 12 weeks for which they underwent PNIF and completed rTNSS/NOSE questionnaires. Cytokine levels were measured by Biolegend FACS with Luminex software used for analysis. The cytokines measured included IL-4, IL-5, IL-6, IL-10, IL-13, IFN-g, and TNF-a.

Symptom changes were evaluated using the paired Wilcoxon signed-rank test. We evaluated Minimal Clinically Important Differences (MCID), which has been defined as 30% of the maximum rTNSS, 24 points in the NOSE scale *or* a 30% reduction in scores, and 20 l/min for PNIF.^13,14^ Exploratory cytokine analyses included Pearson correlation of ΔCytokines with ΔNOSE, linear regression, robust regression (M-estimation), and partial correlation adjusting for baseline NOSE. Analyses were conducted in R version 4.3. Claude AI (Anthropic) was used to assist with manuscript editing and text revision.

## Results

**Figure 1.**
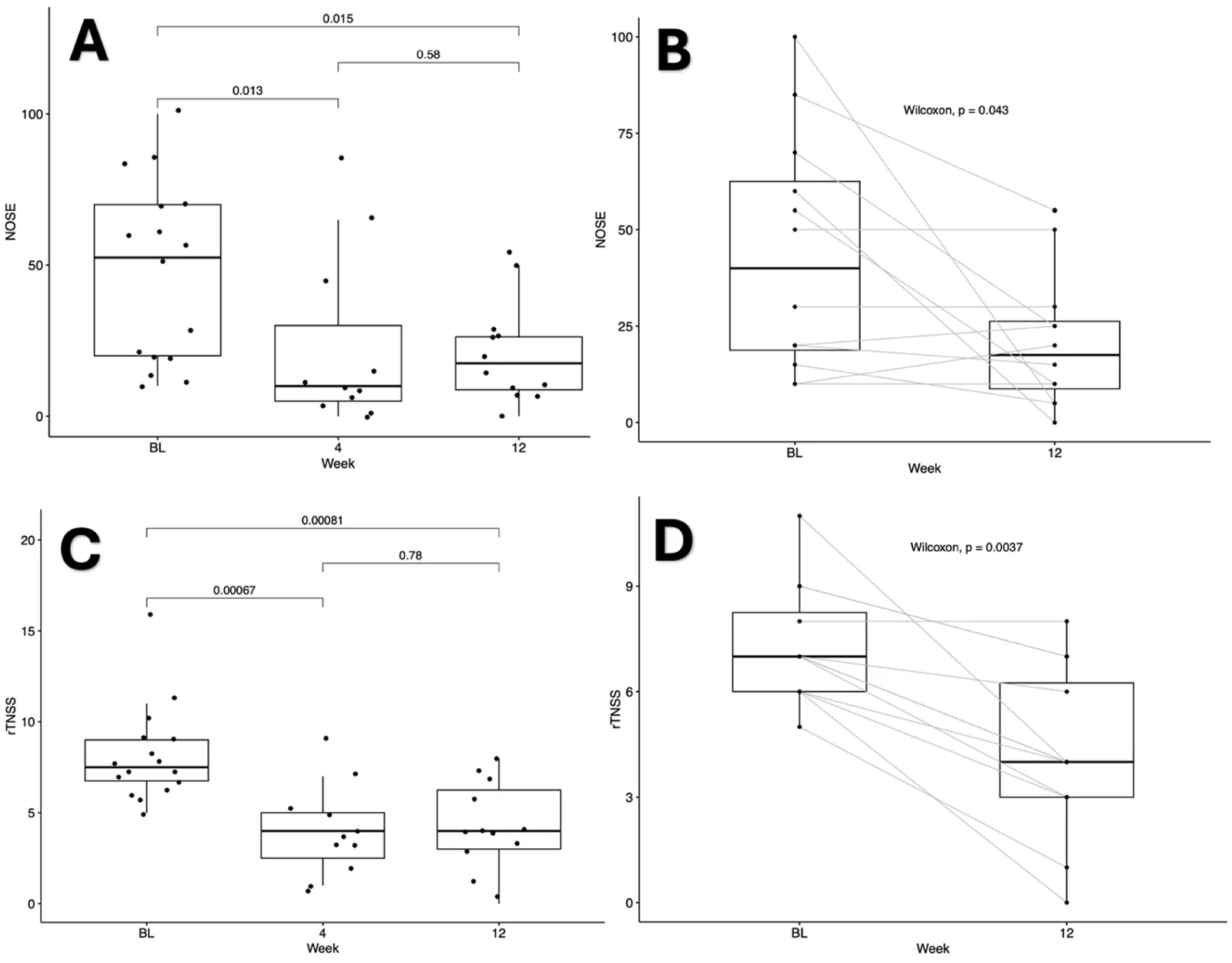
(A) Boxplot with the NOSE scores of the patients in this cohort trended by week. *P*-values reported within the graph. (B) Paired boxplot with the NOSE scores of the patients within this cohort between baseline and post-treatment week 12. (C) Boxplot with the rTNSS scores of the patients in this cohort trended by week. *P*-values reported within the graph. (D) Paired boxplot with the rTNSS scores of the patients within this cohort between baseline and post-treatment week 12. Abbreviations: BL, baseline.

This trial was conducted between January 1, 2023, and February 2, 2024. Fifteen patients underwent this procedure including 8 females and 7 males, with an average age of 61.7 years. The baseline median NOSE score was 52.5 (27.5-77.5 IQR), the median rTNSS score was 7.5 (6.4-8.6 IQR) and the median maximum PNIF was 90 (66.6-113.4 IQR, Table 1 and Figure 1). At the first post-treatment visit, the median NOSE score was 10 (0-22.5 IQR), an 80% decrease (*P* < .05) and the median rTNSS score was 4.0 (2.4-5.6 IQR), a 47% decrease (*P* < .05, Table 1 and Figure 1). The median maximum PNIF at this visit was 85 (46.9-123.1 IQR), a non-significant 5.5% decrease (*P* > .05).

**Table 1.** Table Depicting the Median Values in rTNSS, NOSE, Cytokine Changes, and Average Maximum PNIF Values at Baseline (Pre-Treatment), 4 weeks Post-Treatment and 12 Weeks Post-Treatment.

Values	Baseline (n = 15)	4-wk follow-up (n = 14)	12-wk follow-up (n = 12)	*P-*value
Total rTNSS	7.5 (6.4-8.6)	4.0 (2.8-5.3)	4.0 (2.4-5.6)	<.01
Total NOSE	52.5 (27.5-77.5)	10 (0-22.5)	17.5 (0-28.1)	.04
Maximum PNIF	90 (66.6-113.4)	85 (46.9-123.1)	85 (58.1-111.9)	>.05
Exercise intolerance (NOSE)	6.6	N/A	1.4	.03
Nasal congestion (rTNSS)	2.3	N/A	1	.02
Sneezing (rTNSS)	1.8	N/A	0.9	.02
IL-4	81,140.6	N/A	69,684.0	.6
IL-5	21,603.9	N/A	15,666.5	.4
IL-13	18,163.6	N/A	19,968.4	.8
IL-6	18,715.7	N/A	23,442.0	.5
IL-10	13,934.9	N/A	20,041.5	.4
IFN-g	62,323.6	N/A	66,729.5	.8
TNF-a	64,437.0	N/A	68,194.2	.8

At the second post-treatment visit, the median NOSE score was 17.5 (0-28.1 IQR), a 65% decrease from the baseline (*P* < .05). At this visit, the median rTNSS score was 4 (2-6 IQR), a persistent 47% decrease from baseline (*P* < .05, Table 1 and Figure 1). The symptoms that were reduced at week 12 were exercise intolerance (NOSE), nasal congestion and sneezing (rTNSS, all *P* < .05, Table 1). Importantly, 50% of patients achieved their NOSE MCID (n = 6) while 67% achieved their rTNSS MCID (n = 8).

### Cytokine Analysis

**Figure 2.**
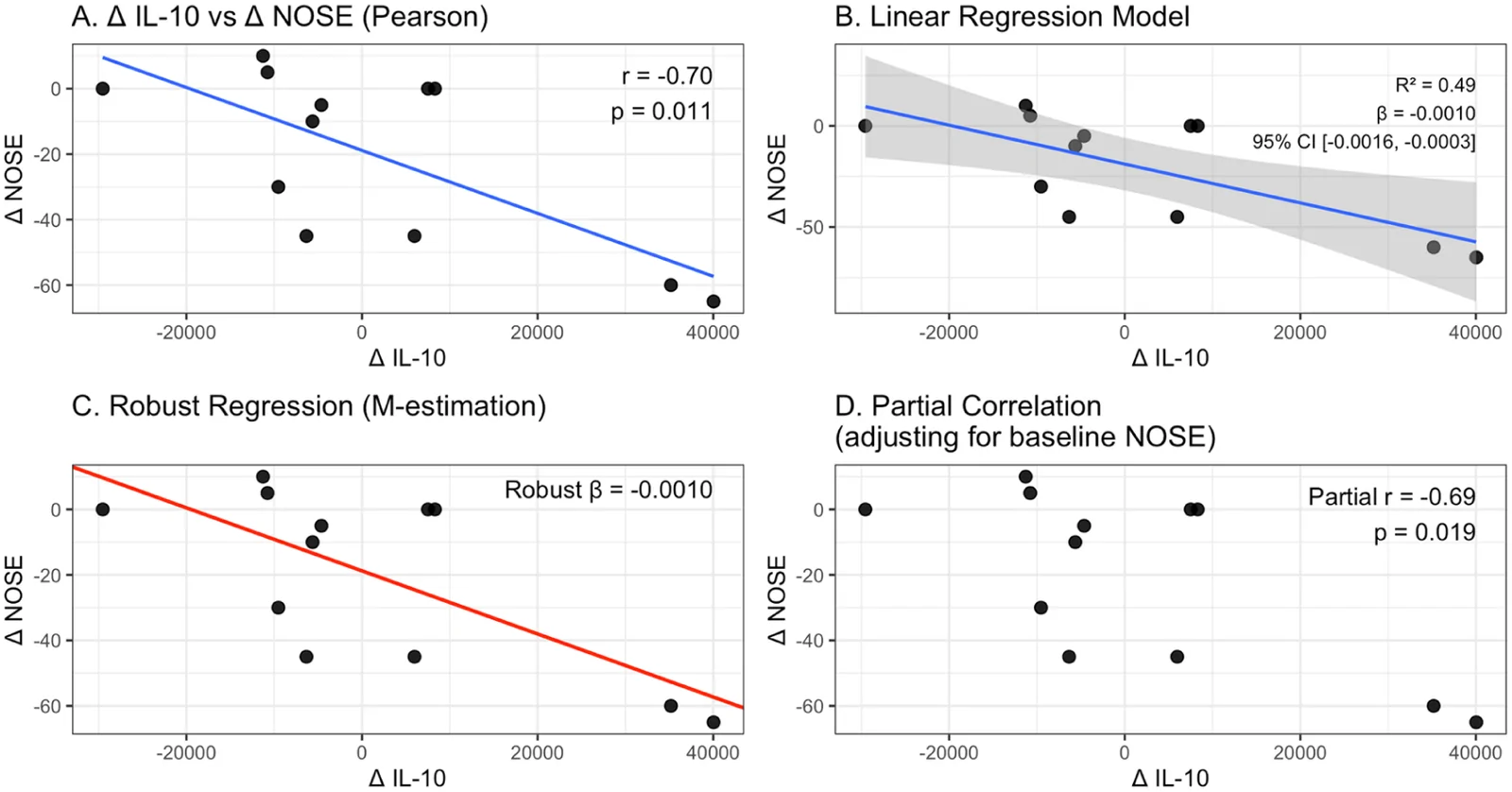
Multimodal evaluation of the relationship between IL-10 change and symptomatic improvement after RFN. (A) Pearson correlation showing that greater reductions in IL-10 were associated with larger improvements in NOSE scores. (B) Linear regression model demonstrating a significant negative association between ΔIL-10 and ΔNOSE, with corresponding β-estimate and *R*^2^. (C) Robust regression (M-estimation) confirming the stability of this association after down-weighting outliers. (D) Partial correlation showing that the relationship between ΔIL-10 and ΔNOSE remains significant after adjusting for baseline NOSE.

Group-level concentrations of all measured cytokines (IL-4, IL-5, IL-6, IL-10, IL-13, IFN-γ, and TNF-α) did not change significantly from baseline to 12 weeks post-treatment (all *P* > .05; Table 1). This is an important negative finding given the study’s aim of identifying objective biomarkers of treatment response. However, patient-level changes revealed an association between IL-10 and symptomatic improvement. ΔIL-10 correlated strongly with ΔNOSE (Pearson *r* = –0.70, *P* = .011; 95% CI −0.91 to −0.21, Figure 2). In linear regression, ΔIL-10 explained 49% of the variance in NOSE improvement (β = –.0010 ± .00031, *P* = .011; *R*^2^ = 0.49, Figure 2). Robust regression confirmed this relationship (*P* = .018). A partial correlation adjusting for baseline NOSE also remained significant (*r* = –0.69, *P* = .019), indicating an independent association between IL-10 changes and symptoms (Figure 2A-D). No other cytokines demonstrated significant correlations.

## Discussion

Establishing an accessible in-office procedure for treatment of rhinitis is important to patients’ well-being and the reduction of medication costs. Additionally, being able to objectively and reliably measure rhinitis severity in a way that incorporates symptoms and science is crucial to our understanding of the pathophysiology. Our results demonstrate a reduction in rhinitis symptoms as measured by rTNSS/NOSE scores after RFN treatment, correlating with prior research.^6^ This study also found that PNIF, a rhinitis-associated marker, did not change after treatment. PNIF stability despite symptomatic improvement suggests that RFN targets neurogenic hypersecretion and mucosal sensitivity rather than anatomical obstruction. This distinguishes RFN as a treatment for secretory symptoms instead of physical airway blockage. Furthermore, while this investigation was limited by a small cohort size, it demonstrates the feasibility of utilizing Leukosorb assays for objective cytokine monitoring during in-office procedures. As such, this study should be interpreted as an exploratory, hypothesis-generating investigation rather than a definitive mechanistic study.

Importantly, the local cytokine environment in the nasal mucosa is of great interest to the rhinology community. Prior research suggests that the local mucosal milieu has a significant impact on rhinitis severity and can serve as a marker for clinical improvement.^12,15,16^ By performing RFN on the PNN, we aimed to modulate the local neuro-immune axis, which regulates glandular hypersecretion and vascular permeability. However, within our study many baseline cytokine levels were at or below the assay’s lower limit of detection, which may have limited the sensitivity for detecting biologically meaningful changes. As a result, the absence of significant group-level cytokine changes may reflect methodological limitations of the Luminex assay in this population rather than a true lack of immunologic effect. As such, individual cell counts by flow cytometry were used for analysis. Despite these limitations, exploratory analyses identified greater reductions in IL-10 correlated with larger reductions in NOSE scores after treatment (Figure 2). This relationship persisted across multiple analytic approaches, supporting a potential biologic association, though this requires validation in larger cohorts (Figure 2). While IL-10 is understood as an anti-inflammatory cytokine, prior literature has demonstrated that its levels can reflect local regulatory mucosal responses, including in the context of immunotherapy.^17^ Further, Benson et al (2000) found that treatment of AR with intra-nasal glucocorticoids reduced IL-10 levels in children.^18^ Overall, at this time, it remains unclear if cytokine changes occur with RF ablation of PNN. This is an important avenue of investigation as cytokine changes in rhinitis could ultimately lead to improved and targeted treatment paradigms.^16,19^

There were no serious adverse events associated with this treatment, and minor side effects reported after included headache, epistaxis, and irritation. The major limitations to this study were the small cohort, PNIF variability, and short follow-up.
